# Synthesis and Modification by Carbonization of Styrene–Ethylene Glycol Dimethacrylate–Lignin Sorbents and their Sorption of Acetylsalicylic Acid

**DOI:** 10.3390/ma13071761

**Published:** 2020-04-09

**Authors:** Krystyna Wnuczek, Beata Podkościelna, Magdalena Sobiesiak, Łukasz Szajnecki, Marta Goliszek

**Affiliations:** Department of Polymer Chemistry, Institute of Chemical Sciences, Faculty of Chemistry, Maria Curie-Skłodowska University in Lublin, M. Curie-Skłodowska Sq. 2, 20-031 Lublin, Poland; magdalena.sobiesiak@poczta.umcs.lublin.pl (M.S.); l.szajnecki@umcs.pl (Ł.S.); marta.goliszek@poczta.umcs.lublin.pl (M.G.)

**Keywords:** lignin, EGDMA, polymer microspheres, sorbent, carbonization

## Abstract

This paper deals with the synthesis and studies of new polymer microspheres properties based on ethylene glycol dimethylacrylate (EGDMA), styrene (St), and various quantities of commercial kraft lignin (L). In the first stage of the investigations, the conditions of the synthesis process were optimized by selecting a proper amount of poly (vinyl alcohol), which was a suspension stabilizer. Next, based on EGDMA + St + L, new polymers were synthesized by the suspension polymerization method. The chemical structure of the materials was confirmed by means of the Attenuated Total Reflectance—Fourier Transform Infrared (ATR-FTIR) analysis. The evaluation of the synthesized materials includes susceptibility to swelling in solvents of different character (polar and nonpolar), porous structure of microspheres, and their thermal resistance. Morphology has been specified by the scanning electron microscope and automated particle size, as well as shape analyzer. The obtained pictures confirmed the spherical shape of the materials. The microspheres porosity was characterized using the low-temperature nitrogen adsorption. To increase the porosity (partially blocked by the large lignin molecule), the microspheres (EGDMA + St + 4L copolymer) were impregnated with the aqueous solution of the activating substance (sulphuric acid, nitric acid, phosphorous acid, and silver nitrate) and then carbonized at 400 °C. After the carbonization process, the increase in the specific surface area was observed. The microspheres were porous with a specific surface area up to 300 m^2^/g. The materials had a desirable feature for their potential use in chromatography, which was confirmed by the results of GC analysis with the acetylsalicylic acid. These materials are an interesting alternative in the field of more environmentally friendly, ecological, and biodegradable polymeric sorbents in comparison to the commonly applied styrene-divinylbenzene (St-DVB) copolymers.

## 1. Introduction

Aliphatic diols (glycols) are chemical compounds containing two hydroxyl groups [1]. Diols are typical reactants and intermediates in a wide range of reactions of esters, aldehydes, amines, azides, carboxylic acids, ethers, as well as in resin formation [2]. These compounds are often used as raw material in the polymer industry because of their hygroscopicity, noncorrosiveness, high boiling points, and solvent properties. Among the glycols, ethylene glycol (EG), diethylene glycol (DEG), and triethylene glycol (TriEG) are the first three members of a homologous series [3]. Ethylene glycol is representative of this group of compounds. It is mainly used as a raw material in the manufacture of polyester fibers. Ethylene glycol is an odourless, colourless, sweet-tasting, and also a toxic substance. In the industry, it has been used as a component of cooling and brake fluids. It is also widespread as a substrate for the production of polyesters. Moreover, EG is characterized by hygroscopicity, which is perfect for use in fibers treatment, paper, printing inks, leather, and cellophane [4]. The group with the most commonly used glycols also include ethylene glycol dimethylacrylate. Ethylene glycol dimathycrelate is a crosslinking monomer, which is often used in the synthesis of polymeric materials. This monomer is characterized by a hydrophilic and polar character, which allows an affinity for the aqueous phase [5]. Summarizing, ethylene glycols and their polymers are used in an enormous range of products and attract considerable interest in many branches of industry and science [6,7,8,9,10,11,12,13].

In the literature, there are not many reports which have been carried out on the synthesis of polymeric materials for sorption processes based on EGDMA and lignin. That indicates the novelty of the presented work. Yang et al. [14] synthesized an organic/inorganic hybrid adsorbent composed of nano-SiO_2_ modified by vinyltrimethoxysilane as the inorganic phase and EGDMA as the organic phase by the dispersion polymerization method. It has been used as an adsorbent for phenol-containing wastewater removal. In another study, Xu et al. [15] prepared an acrylic composite resin that was modified by calcium lignosulphonate using the solution polymerization, where calcium lignosulphonate and acrylic acid were used as raw materials, and EGDMA as the crosslinker. The authors demonstrated a good adsorption capability of the obtained material to methyl orange with the adsorption capacity of 188.33 mg/g. Feng et al. [16] obtained a porous lignin-containing hydrogel for dye removal via graft copolymerization of the acetic acid lignin and acrylamide, using EGDMA as a crosslinker and H_2_O_2_ as an initiator. The authors showed that increased lignin content in the hydrogel promotes the adsorption of methylene blue. The maximum adsorption capacity was 29.65 mg/g. Wang et al. [17] prepared lignin-based encapsulating fertilizers in the form of hollow microspheres for controlled release. The encapsulation efficiency was 46% of the trapped urea as encapsulated inside the lignin microspheres.

One of the methods to prepare porous materials is a pyrolytic reaction called carbonization. During this process, many different reactions concurrently occur (dehydrogenation, hydrogen transfer, isomerization, and condensation). As a consequence of chemical bonds breakdown, some functional groups are lost, while other bonds undergo transformation to form aromatic systems [18]. The presence of aromatic rings in the starting material promotes this process. For this reason, it is desirable for the carbonized material to have as many such structures as possible. Substrates used in this process can be both synthetic materials (polymers) and raw materials of natural origin such as nut shells, fruit stones (e.g., peaches) [18], used industrial byproducts [19], or even wastes from households (coffee grounds) [20]. Plant-based products contain some amounts of lignin which, being rich in aromatic subunits, is a good precursor to carbonization. 

Among different techniques for the treatment of aqueous solutions, which are contaminated with heavy metal ions such as ion exchange, membrane filtration, and chemical precipitation, biosorption is a potential and interesting alternative. It is related to the limitations of the listed techniques, including inefficiency at low concentrations of metal, high cost when treating big amounts of water, and generation of large quantities of sludge and various toxic products that require careful disposal [21,22,23]. Biosorption has superior advantages due to its environmental friendliness and also cost effectiveness. The use of the nonconventional biosorbents, which are prepared from the byproducts and agricultural waste leads to the reduction of the large amounts of solid waste [24]. It is also very attractive because of the availability of waste bioproducts and biomass [25]. This subject is widely studied in the literature. For example, Budnyak et al. [26] synthesized efficient sorbents from technical lignins and silica, which were used for the removal of Methylene Blue dye from the aqueous solution. Obtained materials extracted 80%–99% of the dye in a pH range from 3 to 10. Lapo et al. [27] studied the Hg(II) and Pb(II) removal in single and binary component systems into chitosan-iron(III) biocomposite beads. The sorbent material has more affinity to Hg(II) rather than Pb(II) ions, the maximum sorption capacities were 1.8 and 0.56 mmol/g for Hg(II) and Pb(II), respectively. Klapiszewski et al. [28] prepared and characterized novel TiO_2_/lignin and TiO_2_-SiO_2_/lignin hybrids and used them as functional biosorbents for Pb(II). The experimental adsorption capacities for TiO_2_/lignin and TiO_2_-SiO_2_/lignin were compared and were found to be 35.70 and 59.93 mg/g, respectively. 

A beneficial solution in the synthesis of sorption material is the use of cheap, easily available, and variable raw material. Biopolymers exhibit a great potential as alternatives to classic organic materials because of their biodegradability, biocompatibility, and low production cost. Biopolymer materials have many applications [28]. The example of biorenewable polymer is lignin that can be used for the polymerization process of environmentally friendly materials synthesis [29]. Lignin was introduced to the polymer materials in order to add functionality as it contains various functional groups such as phenolic, hydroxyl, carbonyl, carboxylic, and methoxyl ones in its structure. The presence of these groups makes lignin useful in sorption technologies. The lignin sorption mechanism is not fully understood. Some studies point out to the surface adsorption or ion exchange and several processes proceed simultaneously. Moreover, it is an abundant, inexpensive, and renewable waste material characterized by a largely aromatic structure [30,31,32,33,34,35,36,37,38,39,40].

The paper presents an overview of the composition and properties of polymeric microspheres based on EGDMA, styrene, lignin, as well as the procedure of their carbonization process. The overall performance of polymer materials is described by the swellability, particle size, porous, and thermal properties. Chemical structures of new copolymers have been detected using the attenuated total reflectance Fourier transform infrared spectroscopy (ATR-FTIR). Compared to our previous materials based on divinylbenzene (DVB) and styrene (St), the copolymers based on EGDMA are characterized by a larger amount of lignin introduced into the structure of microspheres. This is due to the fact that the solubility of lignin in EGDMA is higher than in DVB, therefore, EGDMA is more polar and has greater affinity for the functional groups in lignin (phenolic and hydroxyl). It allows increasing the functionalization of these materials, simultaneously increasing their ecological attractiveness. In these studies, the starting material for carbonization is the polymer containing in its structure aromatic units derived from styrene and a small amount of lignin. As a product of heat treatment porous carbon microspheres are obtained. In order to receive a higher porosity of the microspheres, they were subjected to a carbonization process under various conditions. The carbonized materials were examined as a sorbent for the chemical substance (acetylsalicylic acid), the component of the popular drug aspirin, which is the pharmaceutic belonging to the group of nonsteroid, antiphlogistic drugs. The sorption capacity was tested by the GC analysis. As follows from the measurements of the acetylsalicylic acid concentration the EGDMA + St + L based carbonized copolymers can be used as sorbents.

## 2. Materials and Methods 

### 2.1. Chemicals and Eluents

Kraft lignin was obtained from Sigma-Aldrich (Steinheim, Germany). Poly (vinyl alcohol) (APV) M_w_ = 72000, 98% degree of hydrolysis, benzyl alcohol, styrene (St), and ethylene glycol dimethylacrylate (EGDMA) were obtained from Merck (Darmstadt, Germany). α,α’-Azoiso-bis-butyronitrile (AIBN) was obtained from FLUKA (Buchs, Switzerland). Toluene (TOL), decan-1-ol, tetrahydrofuran (THF), acetone (Ace), chloroform (TCM), methanol (MeOH), and acetonitrile (ACN), were obtained from Avantor Performance Materials Poland S.A. (Avantor, Gliwice Poland). Purified water came from Millipore (UMCS Lublin, Poland). The sulphuric acid, nitric acid, phosphorous acid, silver nitrite, and caffeine came from Merck (Darmstadt, Germany). The acetylsalicylic acid (ASA) was from Sigma-Aldrich (Darmstadt, Germany).

### 2.2. Synthesis of EGDMA-Based Polymeric Microspheres

In the first stage, the optimization amount of poly (vinyl alcohol) which was a suspension stabilizer was carried out. The various amounts of APV from 1 to 5 g were used. As the amount of APV increased, the microspheres became smaller, 2 g of APV for 150 mL of water was considered the optimal amount. 

**Figure 1:**
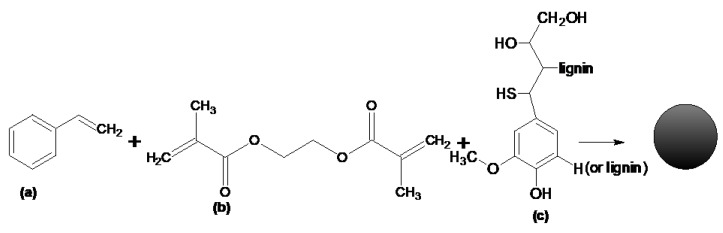
Chemical structure of monomers. (**a**) styrene, (**b**) ethylene glycol dimethacrylate, (**c**) lignin.

Different quantities of kraft lignin (Table 1) were dissolved in benzyl alcohol (14 mL). The process of dissolving lignin lasted 24 h, in a static manner at room temperature. The APV (1 g) and purified water (75 mL) were placed in a 250 cm^3^ round-bottomed flask equipped with a mechanical stirrer and a thermometer. The mixture was stirred intensively to dissolve the suspension stabilizer for 0.5 h at 80 °C. Next, the monomers EGDMA and St were added to the dissolved lignin. Finally, the organic mixture was added to the aqueous phase. The copolymerization of the reactive mixture was performed in the aqueous medium in the presence of the initiator. The initiator (AIBN) was added in the amount of 2 wt% of monomers. The reaction mixture was stirred at 300 rpm for 8 h at 85 °C. When the reaction was over, the resulting precipitate was filtered off. In order to remove the APV and solvent (benzyl alkohol), the obtained lignin microspheres were washed several times with distilled hot water (2 L) and finally purified with acetone in the Soxhlet’s apparatus (6 h). After drying, the microspheres were fractionated with sieves. The applied polymerization conditions and different quantities of lignin yielded microspheres with various diameters. Figure 1 presents the chemical structures of the monomers used for the synthesis of polymeric microspheres.

### 2.3. Characterization of Methods

The attenuated total reflectance (ATR) with the Fourier transform infrared (FTIR) spectra of all samples was recorded using the Bruker TENSOR 27 FTIR spectrophotometer (Ettlingen, Germany). There were 32 scans accumulated in the range of 600–4000 cm^−1^. The spectrophotometer was equipped with a diamond crystal.

Scanning electron micrographs were collected from Quanta 3D FEG (FEI) (Hillsboro, OR, USA). Prior to the analysis, the samples were coated with a 3-nm thick gold layer.

Morphological analysis of the particles of the examined sorbents was carried out by the automated morphology particle size and shape analyzer (Morphologi G3, Malvern) microscope. Before the measurements, the particles of the analyzed samples were dispersed using a sample dispersion unit (SDU) with the following parameters: Injection pressure = 2.0 bar; injection time = 40 ms; settling time = 120 s. The measurements were performed on the 16 cm^2^ area.

Describing the size and shape of the three-dimensional (3D) particle is a complex issue, particularly in the case of nonspherical or irregular particles [41]. The analysis captures a two- dimensional (2D) image of a 3D particle and calculates various sizes and shape parameters from this 2D image. These parameters allow for quantifying characterization of the particle morphology [34]. The major axis (M_Ax_) of the 2D projection of the 3D particle passes through the centre of mass (P_CoM_) of the particle shape at an orientation corresponding to the minimum rotational energy of the shape. The length of the particle (P_L_) is defined as the longest line between two points on the perimeter of the 2D projection of a particle on the plane projected onto the major axis whereas the particle width (P_W_) is the longest line between two points on the perimeter of the particle projection projected onto the axis perpendicular to the major axis. Another way to describe the particle size is the circle equivalent diameter (CE_diam_), which is defined as the diameter of a circle with the same area (C_A_) as the projected area of the particle image (P_A_). This is a single number value that gets information about the size and allows comparing the size of two particles with different shapes. Very differently shaped particles may be characterized as identical using only CE_diam_ because they have similarly projected 2D areas. Thus, the information about particle shapes is required. Figure S1 presents the 2D sketch of the 3D particle and circles the equivalent idea. The important parameters used for describing the particle morphology are presented in Supplementary Information (SI) (S2.3. Characterization of Methods).

The swellability of coefficient B was determined by the equilibrium swelling in acetone, methanol, acetonitrile, chloroform, toluene, THF, and water using the method proposed by Tuncel and Pişkin where B is expressed as [42]:

(1) $$B=\frac{V_{s}-V_{d}}{V_{d}}\;\times \;100\%$$

where *V_s_* is the volume of the copolymer after swelling and *V_d_* is the volume of the dry copolymer.

Measurements were made using a semi-permeable membrane tube. One tenth g of the materials was added to the tubes. The tubes were placed in beakers with various solvents (10 mL). 

Each measurement lasted 30 min. After this time, the volume of materials was examined. The swelling coefficients were calculated according to formula B.

Differential scanning calorimetry (DSC) thermograms were performed with the use of a Netzsch 204 calorimeter (Netzsch, Günzbung, Germany). All DSC measurements were obtained in the aluminum pans with pierced lids with a sample mass of ~5–10 mg in the nitrogen atmosphere (30 mL × min^−1^). Dynamic scans were performed at a heating rate of 10 K × min^−1^ in the temperature range of 20–500 °C.

Thermal analysis was carried out on a Netzsch, STA 449 Jupiter F1 (Selb, Germany). The samples were heated from 30 to 800 °C at a rate of 10 °C × min^−1^ in the dynamic atmosphere of helium (25 mL × min^−1^). The sensor thermocouple type S TG–DSC was used with the empty Al_2_O_3_ crucible as the reference. The loss mass temperatures (T_5%, 10%, 50%_), peak maximum decomposition temperatures (T_max1, max2_), and residual mass at 800 °C (RM) were determined.

The carbonization process took place in an electric furnace. The temperature during the process was 400 °C. The process was carried out for 0.5 h. Crucibles with the polymer material in an amount of about 2 g were placed in a quartz tube. The carbonization process was carried out in a continuous flow of nitrogen. The remains of the polymeric sample were divided into five portions (ca. 4.5 g) and each of them was treated with a different activating agent. The aqueous solutions of 85% phosphoric acid (diluted 1:1 v/v (P2) and undiluted (P1)), 95% sulphuric acid (diluted 1:1 v/v (S)), 65% nitric acid (diluted 1:1 v/v (N)), and silver nitrate (9% (Ag)) were used as surface activators. The polymer samples were soaked in the solutions for 24 h and then dried in the air at 40–50 °C. The thoroughly dried samples were weighed (2–2.5 g) into crucibles and placed in the electric furnace. As previously, the carbonization process was conducted in the nitrogen atmosphere for 30 min at 400 °C. In the names of the prepared materials the type of activator was marked with the symbol of the characteristic atom.

The specific surface area of the microspheres was determined from the low-temperature nitrogen adsorption data (automatic sorption analyzer ASAP 2420, Micrometrics, (Norcross, GA, USA). Prior to the measurements all samples were degassed at 120 °C (for 2 h). The value of the Brunauer–Emmett–Teller (BET) specific surface area was calculated automatically by the equipment. All the pore volumes and pore size distributions were determined by the Barrett–Joyner–Halenda (BJH) method.

All chromatographic analyses were performed by a gas chromatograph (GC) with an FID detector (GC-2010, Shimadzu, Japan). Potential sorption properties of the studied materials after carbonization were examined. The extent of the decrease of the tested substance (acetylsalicylic acid) concentration in the solution above the sorbent compared to that of the starting solution was taken as a measure of carbonization sorption capacity. At the beginning, 0.2 g of selected polymeric material was weighed for a vial. Each vial was flooded with the 1.5 mL standard solution of ASA (the initial concentration of ASA in methanol was 500 μg/mL). The vials were capped and shaken for 30 min. The next step was centrifugation (2500 rpm for 3 min). Supernatants were filtered through a syringe filter (0.45 μm). Twenty μL of caffeine solution (as an internal standard) was added to the filtrate. Gas chromatography analyses were conducted. The ASA concentration in the blank sample (without any sorbent) was also determined. The measurement procedure was applied three times for each sorbent. The average value and standard deviation were calculated for each sample.

## 3. Results and Discussion

### 3.1. Characterization of the EGDMA + St Microspheres with a Lignin Component

**Figure 2:**
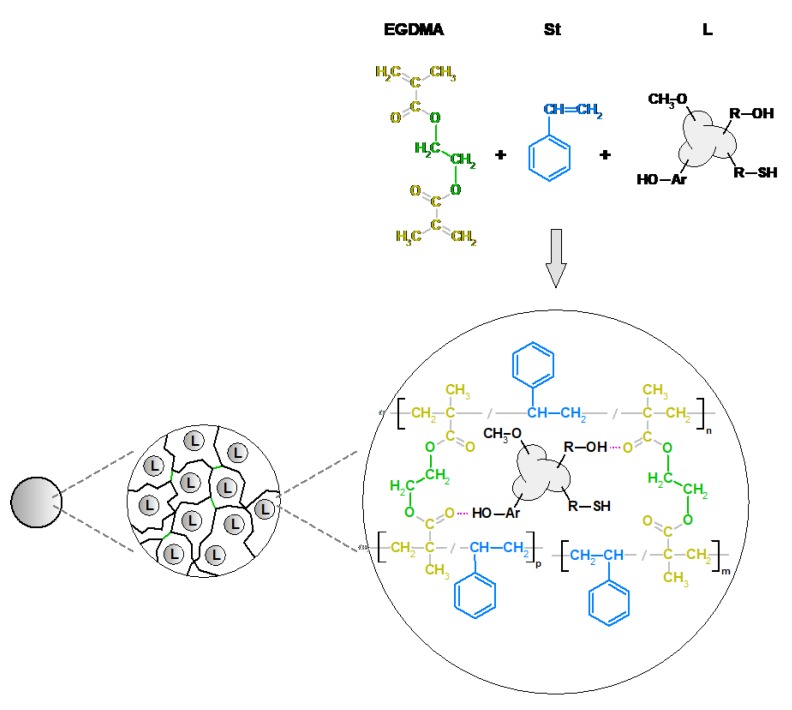
Possible chemical structure of polymeric microspheres.

**Figure 3:**
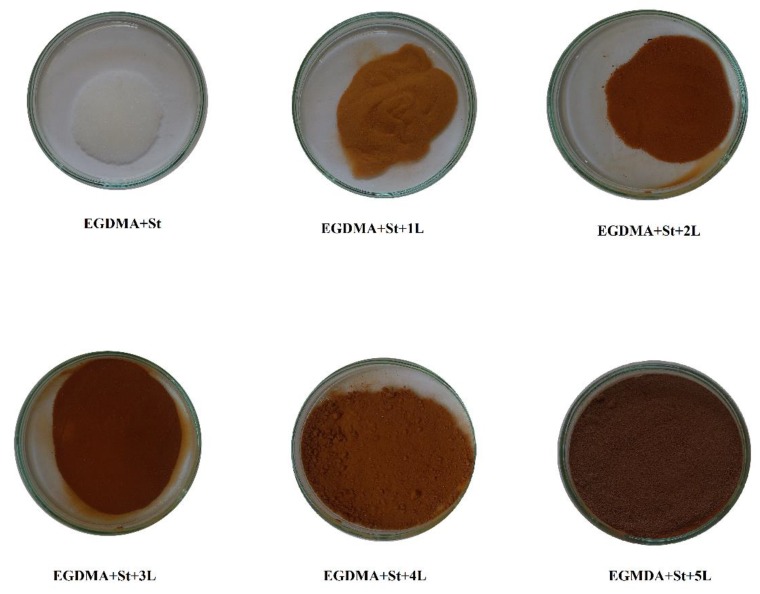
Photos of the obtained microspheres EGDMA-St with different amounts of lignin (1–5 g).

Figure 2 shows the possible structure of the copolymer EGDMA + St with lignin. Three components were used to prepare polymeric microspheres. Each of the monomers performed a definite function during the formation of polymer microspheres. Ethylene glycol dimethylacrylate was used as a crosslinking monomer. This process makes microspheres resistant to the mechanical damages and action of solvents. EGDMA is a popular monomer used for the preparation of methacrylate composites. Styrene is an aromatic, very reactive, active solvent. The last component is the commercial kraft lignin. The presence of polar groups in the lignin structure increases the chemical affinity of this biopolymer for the EGDMA. In this way, a high percentage of lignin in the copolymer structure reaches the 90% yield (Table 1). Moreover, the presence of the functional groups in its structure modifies the surfaces of the microspheres. Unfortunately, lignin attaches to the microspheres on their outer part and thus blocks the pores. Therefore, the obtained microspheres are characterized by a low porosity. Due to the fact that lignin is a biopolymer that contains a lot of aromatic parts in its structure, the carbonization process of the microspheres was induced in order to increase their potential applications. After the carbonization process, the porosity of polymeric microspheres expanded. Characterization of the carbonized materials was made as compared to the polymeric precursor EGDMA + St + 4L, which will be discussed in the next part of the article. In Figure 3, photos of the obtained microspheres are presented. As one can see, as the amount of lignin increases, the microspheres become darker.

### 3.2. Spectroscopic Characterization of Copolymers

**Figure 4:**
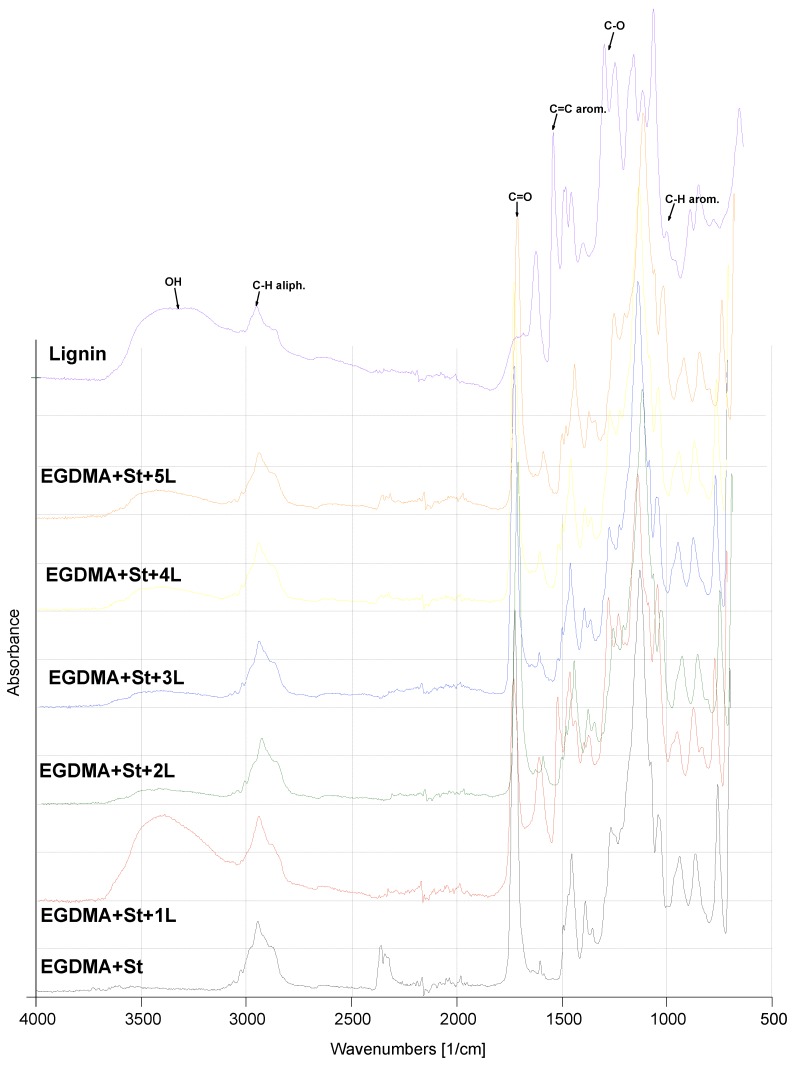
Attenuated Total Reflectance/Fourier Transform Infrared spectra of lignin and copolymers.

The first product was obtained from the reaction of EGDMA with St. This system was chosen as the reference. The ATR-FTIR results are shown in Figure 4. The exact values of the wave numbers are also given in Table 2. In Figure S2, the ATR-FTIR spectra of monomers and copolymer (EGDMA-St) is visible. In the EGDMA and St spectra, the signal corresponding to C=C stretching vibration of vinyl group (1640 cm^−1^) is clearly noticed. The lack of this signal in the EGDMA-St spectrum indicates the correct course of the copolymerization reaction. Changes in the absorbance of some signals were observed in the spectrum as the amount of lignin increased. This effect was observed for the adsorption bands around 3400–3500 cm^−1^, which causes the appearance of a hydroxyl group from the lignin molecule to be visible. Additionally, in Figure S3, the ATR-FTIR spectra in the range 2000–4000 cm^−1^ of –OH groups in copolymers and original lignin are shown. With the addition of lignin amount, the changes in the intensity of signals are visible. Due to the increase in the color intensity of the microspheres, this growth in signal intensity is not directly proportional to the amount of lignin (impact of the ATR-FTIR method). Nevertheless, the general trend (increase of intensity of –OH groups) is maintained. Ring vibrations are visible around 1600 cm^−1^ and come from the aromatic lignin part (Figure 2). The more lignin in the polymeric material, the lower are the signals from the aliphatic part. Both the spectra of EGDMA + St + L copolymers and pure lignin contain the band from C=C and C–O. Moreover, the spectra of EGDMA + St + 2,3,4L look very similar. The original lignin exhibited a wide absorption band at 3400–3200 cm^−1^ indicating the presence of OH stretching vibrations in the aliphatic and aromatic hydroxyl groups. 

### 3.3. Morphology of EGDMA + St + lignin Microspheres

**Figure 5:**
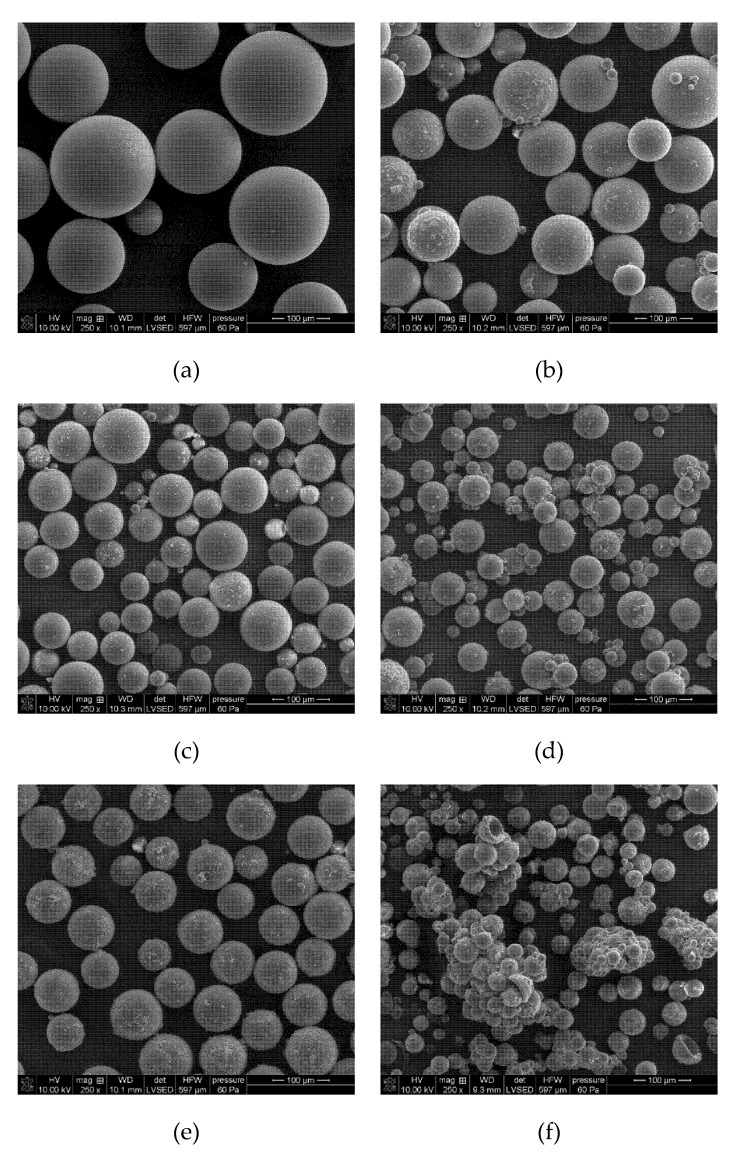
SEM images of the obtained microspheres. The scale bar is the same for all images. (**a**) EGDMA + St, (**b**) EGDMA + ST + 1L, (**c**) EGDMA + St + 2L, (**d**) EGDMA + St + 3L, (**e**) EGDMA + St + 4L, (**f**) EGDMA + St + 5L.

Figure 5 shows the pictures of the obtained sorbents. The SEM pictures confirmed the spherical shape of molecules. For the free-lignin copolymer the highest circle equivalent diameter is observed. The increase of the lignin amount in the sample includes the reduction of diameter. The diameter reduction based on the added value of lignin was confirmed in Figure 5. The EGDMA + St copolymer is characterized by the highest surface homogeneity, with the addition of lignin reducing this feature (more irregularity), which is visible on the surface of microspheres. The analysis of SEM images shows that the sorbent with the highest lignin content (5 g) contains agglomerates (Figure 5f). The sorbent with 4 g of lignin was chosen as the representative material for the carbonization process due to its homogeneous particles (Figure 5e).

The exact quantitative description of the parameters was made using the optical microscopy measurements: Morphology G3 particle size and shape analyzer. The obtained results confirm the conclusions drawn from the SEM pictures. Table 3 shows the results of morphological characterization of sorbents. The microsphere diameter for EGDMA-St copolymers is approximately 130 μm, while after adding lignin it decreases within 28–53 μm. Analyzing the given parameters, it was found that the free-lignin sorbent is characterized by the highest parameter of the circle equivalent diameter (CE_diam_), circularity (C), and elongation (E). The more lignin in the sample, the roughness increases or the value of the parameter indicates the solidity (S). The EGDMA + St + 5L copolymer has the highest elongation parameter, which indicates that this system is inhomogeneous. Although the system with 4 g of lignin is a polymer with a high content of biopolymer, it still retains a spherical form of the particle. This fact made the EGDMA + St + 4L sorbent the most promising material for the carbonization process.

**Figure 6:**
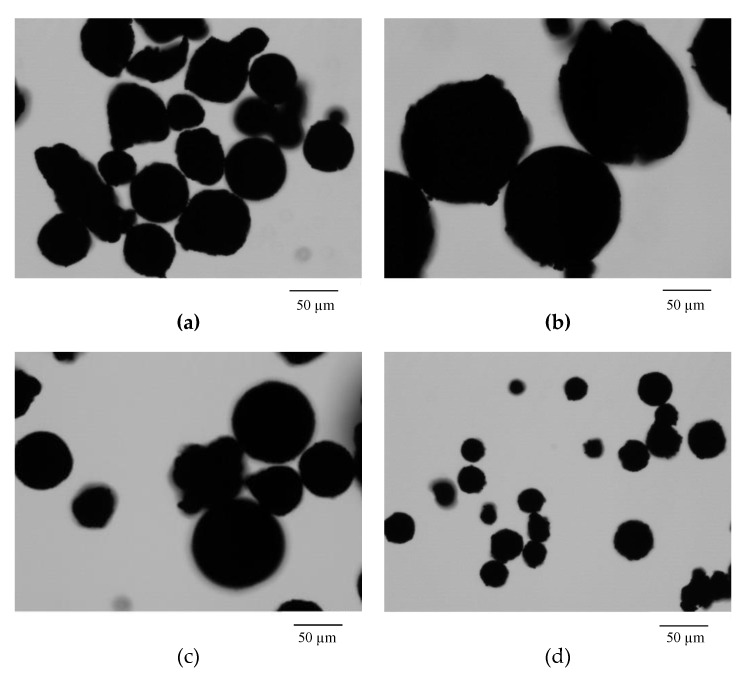
Morphology of carbonized microspheres. The scale bar is the same for all images. (**a**) EGDAM + St + 4L-C, (**b**) EGDMA + St + 4L-P1, (**c**) EGDMA + St + 4L-N, (**d**) EGDMA + St + 4L-Ag.

For the systems EGDMA + St + 4L-C (Figure 6a), EGDMA + St + 4L-P1 (Figure 6b), EGDMA + St + 4L-N (Figure 6c), and EGDMA + St + 4L-Ag (Figure 6d), the microspheres have retained their sphericity. For the rest of the systems, the microspheres have not kept their original spherical shape. The particles were stuck together and agglomerated.

**Figure 7:**
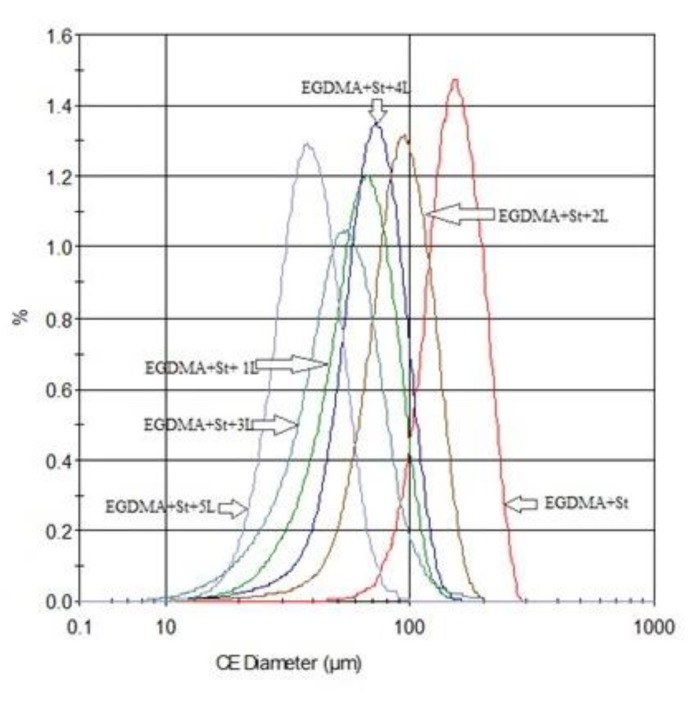
Impact of lignin content on the diameter distribution of microspheres.

Figure 7 shows the distributions of diameters of the analyzed polymers. The particles of the sample EGDMA-St (without lignin) have a higher value of CE diameter. The presence of lignin in the polymers reduces the microspheres diameter. The morphological characterization of sorbents parameters is shown in Table 3. The material of the largest grain size is the initial material EGDMA + St, but the sorbent prepared with the share of the largest amount of lignin (EGDMA + St + 5L) contains the particles of the smallest sizes.

### 3.4. Swelling Studies

Swellability is a factor that defines accessibility of the internal chemical structure in the crosslinked polymers for penetration by solvent molecules. The swellability coefficients in tetrahydrofuran (THF), acetone (ACE), acetonitrile (ACN), toluene (TOL), chloroform (TCM), methanol (MeOH), and water were studied. The results are summarized in Table 4. Based on the results presented below, one can conclude that the highest values of swelling coefficients were obtained for toluene and acetone. Water has the lowest tendency to swell which proves that the obtained copolymers swell more easily under the influence of organic solvents than in water. All of the copolymers with lignin reached the highest swellability coefficient values in toluene. Microspheres swell in toluene, tetrahydrofuran, and acetone. Toluene has the highest swelling coefficient for each copolymer.

Microspheres swell in toluene, tetrahydrofuran, and acetone. Toluene has the highest swelling coefficient for each copolymer except EGDMA-St copolymers without the addition of lignin. This copolymer generally shows very low swelling in all solvents. This is due to the low porosity of this system. Six percent swelling for ACN and THF results from their greater polar nature and greater interaction of these solvents with the polar EGDMA. 

Microspheres have higher affinities for hydrocarbons solvents. The lowest swelling coefficients were shown for the EGDMA + St polymer without lignin, which indicates an increase in swelling after the lignin presence. As the amount of lignin in the microspheres increases, the swelling coefficient increases. The copolymer EGDMA + St + 5L was characterized by the highest susceptibility to swelling. Generally, materials based on EGDMA have a low tendency to swell due to their low porosity. The addition of polar groups from lignin causes an increasing in the interaction of solvents with the microspheres. After carbonization the resulting materials do not indicate a tendency to swell.

### 3.5. Thermal Properties of Copolymers

**Figure 8:**
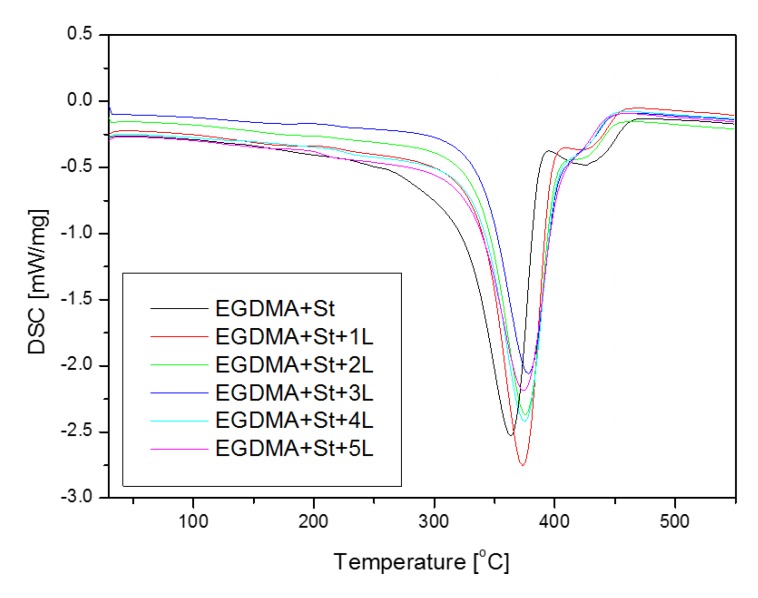
Differential scanning calorimetry curves of copolymers.

Figure 8 illustrates the DSC curves for all obtained microspheres. On the curves of copolymers one can observe two endothermic effects, the first large effect around 360 °C related to the decomposition of the aliphatic part of EGDMA and the second around 420 °C related to the degradation of the aromatic part of St and lignin. For the copolymer with a large amount of lignin (EGDMA + St + 5L) the DSC curve deviates from the trend. It is worth noting that as the amount of lignin increases, the endothermic effect of about 420 °C is not observed which indicates a greater homogeneity of the examined system. With the increases in the amount of lignin in the structure of the microspheres, the slower decomposition takes place (Figure 8). The decomposition EGDMA + St sample, obtained without the addition of lignin, at the lowest temperature is observed. Two exothermic peaks in the range of 300–450 °C can be due to intramolecular crosslinking and repolymerization of the smaller lignin molecules formed via bond cleavage.

**Figure 9:**
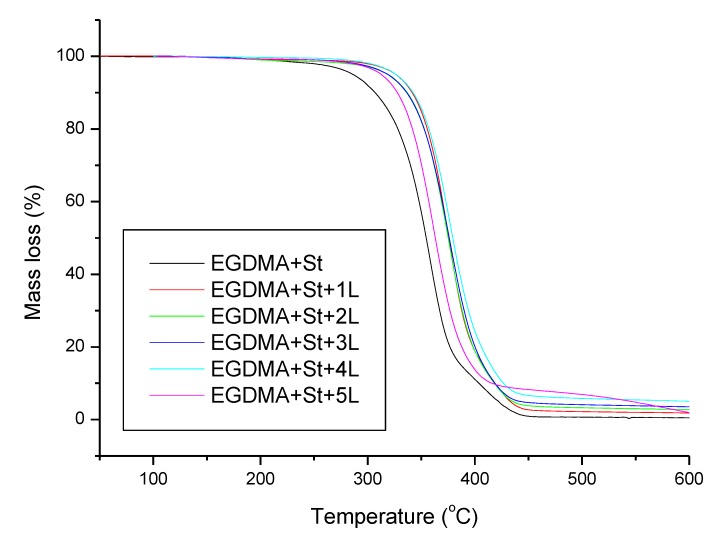
Thermogravimetry curves of the obtained materials.

**Figure 10:**
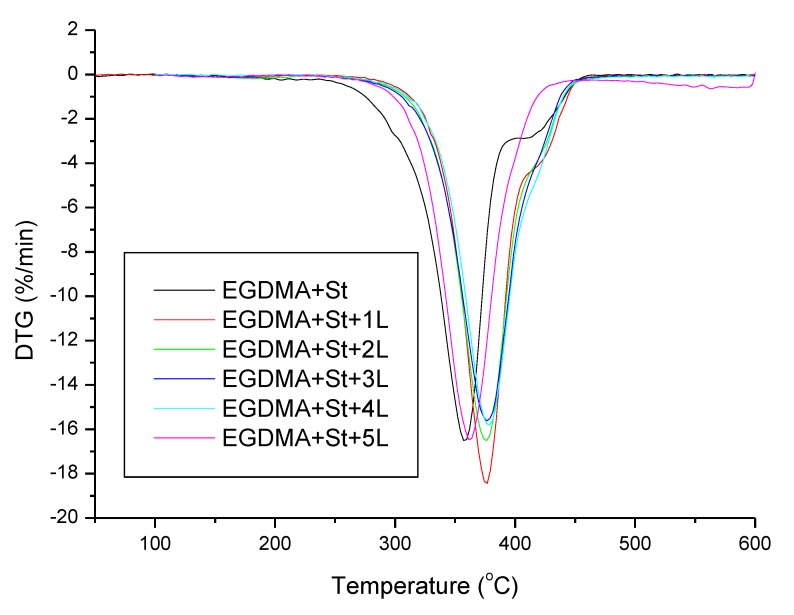
Derivative thermogravimetric curves of the obtained materials.

The thermal stability and degradation behaviour of the obtained materials were studied by means of thermogravimetry. The results are summarized in Table 5. The addition of lignin resulted in higher thermal resistance of the synthesized polymeric microspheres, which can be related to the aromatic structure of lignin. As far as the reference sample without lignin (EGDMA + St) and the sample with 1 g of lignin (EGDMA + St + 1L) are concerned, the thermal degradation proceeded in two steps whereas for the other samples in one step. This observation leads to the conclusion that larger amounts of lignin resulted in homogenization of the material. The residual mass increases with the increasing amount of lignin in the material. The exception to this relationship is the sample with 5 g of lignin (EGDMA + St + 5L) where a smaller amount of residual mass is observed. This may be due to a difficult process of larger, higher amounts of lignin (5 g) incorporation into the microspheres. As follows from the thermal analysis, the obtained microspheres are characterized by good thermal resistance. Figure 9 and Figure 10 present the TG/DTG curves. 

### 3.6. Carbonization Process

For the carbonization process, the copolymer EGDMA + St + 4L was chosen as a representative sample. It was characterized by a large share of lignin and spherical shape without agglomeration tendency. Characteristic of the carbonization process are summarized in Table 6.

At first, the reference material (EGDMA + St + 4L-C) was prepared in the following way: 4 g of the output sample was carbonized for 30 min at 400 °C in the nitrogen atmosphere. No activating agent was used in this case. The yield of carbonization processes was various from 8% (without the activator) to 26% (in AgNO_3_).

The carbonization process showed how the specific surface would be affected by the activator and temperature (Table 7). All the materials obtained by carbonization possessed larger porosity parameters (S_BET_ and V_TOT_) than the output polymeric sample. Depending on the kind of surface activators the specific surface areas ranged from 1 to 299 m^2^/g. In the case of carbons prepared by activation with the phosphoric acid, a larger area was obtained for the diluted acid. Analyzing the results for EGDMA + St + 4L-P2, EGDMA + St + 4L-S, and EGDMA + St + 4L-N it should be noted that the diluted sulphuric acid was the most efficient in the activation process and nitric acid was less efficient, which can be associated with the increase of thermal stability of their molecules. The same relationship is observed when the porosity of EGDMA + St + 4L-N and EGDMA + St + 4L-Ag is compared. The more thermally stable silver nitrate (ionic compound) gives carbon that is characterized by larger porosity. The volatile products of decomposition of an activating agent react with the carbonized polymer. During this process, the oxidation and removal of some fragments of the polymer network takes place, as a result free space in the structure is generated, which forms porosity. The described process is more effective if the starting material is porous. Taking into account that the output sample was nonporous the obtained results seem to be quite satisfactory. 

### 3.7. Porous Structure

The EGDMA + St + L copolymers were characterized by a very small specific surface area (below 1 m^2^/g). As mentioned earlier owing to a large efficiency of introducing unmodified lignin into the structure of polymers, the application of this material as a precursor of the carbonization process was very promising. The surface area and porosity results for the carbonized materials are presented in Table 7. The structural parameters depended on the conditions in the carbonization process. The largest values of the specific surface areas and total pore volumes were found for the copolymer, which was carbonized with sulphuric acid. However, the lowest values were not for the carbonized material. The specific surface areas and total pore volumes are in the ranges of 0.55 to 299.4 m^2^/g and 0.0046 to 0.1492 cm^3^/g, respectively. The results confirm that the carbonized materials can be used as sorbents.

### 3.8. Chromatography Analysis

**Figure 11:**
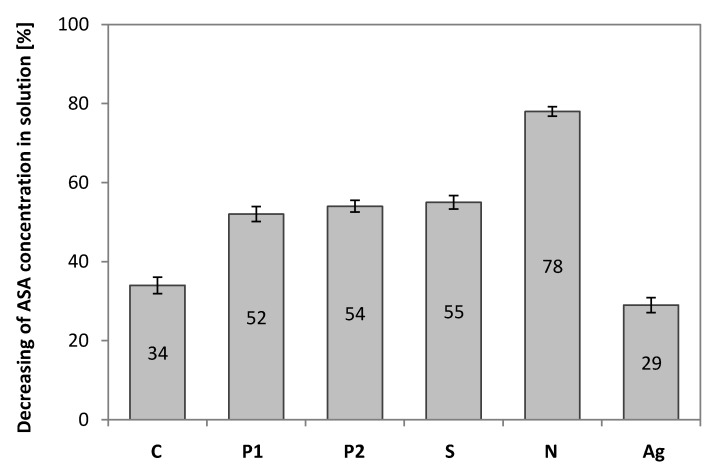
Sorption of Acetylsalicylic Acid on the studied sorbents.

The graph shows the significant influence of the carbonization process for sorption. The greatest influence is demonstrated by nitric acid (75% of the ASA initial amount was adsorbed) and the lowest by the silver nitrite (sorption of ASA amounted to 29%). The adsorption capacities of the carbonized materials are shown in Figure 11.

**Figure 12:**
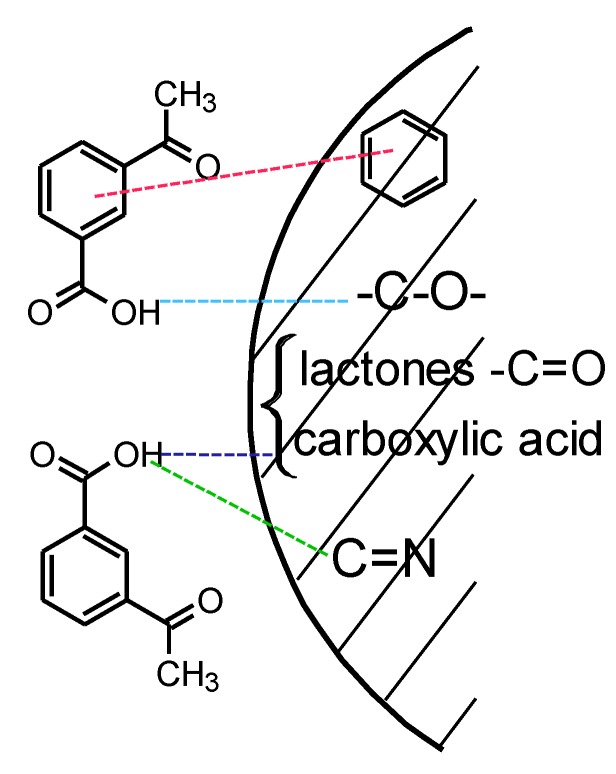
Proposal mechanism of sorption.

The largest ASA sorption has the EGDMA + St + 4L + N copolymer. This carbon has a low specific surface area. The sorption is due to the interactions between the functional groups present on carbon and ASA. In order to determine the chemical characterization of groups present in the carbonized materials, the ATR-FTIR analysis (Figure S4) and pH determination were performed. The pH for EGDMA + St + 4L-N is 8, for EGDMA + St + L-Ag and EGDMA + St + 4L-C is 7, and for the rest within 5–6. The material which is the most effective represents a slightly alkaline character. Analyses for the copolymer EGDMA + St + 4L + N showed that the presence of absorption bands in the area of 1720 cm^−1^ correspond to stretching vibrations of lactones and carboxylic groups. The signal at 1600 cm^−1^ is related to C=N group and about 700 cm^−1^ to out of plane ring deformation. Acetylsalicylic acid has an ester and carboxyl group and an aromatic ring. Therefore, the basic interactions that should be considered are hydrogen bonds (between functional groups) and π–π electron interactions between the aromatic rings of salicylic acid and sorbents and van der Waals interactions. The proposal scheme of sorption is presented in Figure 12 [43].

## 4. Conclusions

As a result of copolymerization reactions of ethylene glycol dimethacrylate with styrene and kraft lignin, new polymeric materials in the form of microspheres were obtained. Replacing the commonly used DVB by EGDMA and the addition of biopolymer—lignin is an interesting alternative in the field of more ecological and biodegradable polymeric sorbents. The ATR-FTIR spectra confirmed lignin incorporation into the structure of microspheres. The increase of intensity of the hydroxyl and aromatic signals from lignin was observed. The swellability coefficient in the typical organic solvents and water for the polymeric microspheres was measured. The greatest tendency towards sorbent swelling was observed for toluene, acetone, and tetrahydrofuran. The free-lignin copolymer exhibited no swelling tendencies. The obtained biopolymer microspheres have a good thermal resistance, the initial decomposition is in the range 360–420 °C. The decomposition process is over at 450 °C.

The results of microscopic studies show that the molecules of the obtained sorbents have a spherical form. With the increasing lignin content in sorbents, the sorbent grain diameters diminish and their form departs from the spherical one. The presence of lignin incorporated into the microspheres structure results in the surface “roughness” increase. The particles of the sorbent with the largest amount of lignin show the tendency towards agglomeration.

One of the polymers containing a relatively large lignin content (EGDMA + St + 4L) was carbonized using various compounds that acted as surface activating agents. Among them, phosphoric, sulfuric acid, and nitric acids, as well as silver nitrate were used as activators. The obtained carbonized materials had specific surfaces in the range of 1 to 299 m^2^/g. Sulphuric acid turned out to be the most effective surface activator.

The effects of carbonization conditions on the sorption properties of the obtained materials were studied. The ability of six samples of carbonized sorbents based on EGDMA + St + 4L to adsorb ASA was investigated. Of all carbonization conditions, the sorbent obtained in the presence of nitric (V) acid is characterized by the largest sorption capacity of ASA. However, using AgNO_3_ as an activator reduces sorption properties of the obtained material below the sorption capacities of the carbonizate obtained without any activator. The presence of functional groups introduced before the carbonization process, using an appropriate promoter, may significantly increase sorption by the presence of interactions between the functional groups of the analyte and carbons (e.g., hydrogen bonds).

## Figures and Tables

**Table 1:** Experimental parameters of microspheres synthesis.

Kraft Lignin [g]	St [g]	EGDMA [g]	AIBN [g]	Yield [%]
0	4	3.8	0.18	74
1	4	3.8	0.20	90
2	4	3.8	0.22	83
3	4	3.8	0.24	80
4	4	3.8	0.26	81
5	4	3.8	0.28	87

**Table 2:** Wavenumbers [1/cm] of characteristic bands visible on Fourier Transform Infrared - Attenuated Total Reflectance spectra.

	C–H aliph.	C–H arom.	C=C arom.	C–O	C=O	–OH
EGDMA + St	2943 1386	941 862	1453	1259 1127	1723	—
EGDMA + St + 1L	2939	941 860	1597	1265 1128	1720	3402
EGDMA + St + 2L	2943	940 861	1599	1263 1127	1722	3434
EGDMA + St + 3L	2939	937 861	1599	1262 1178	1722	3420
EGDMA + St + 4L	—	939 860	1598	1262 1128	1722	3500
EGDMA + St + 5L	2941	937 860	1598	1263 1128	1722	3402
Lignin	2950	930	1620	1300	1680	3510
EGDMA	2951	—	—	1293	1717	—
St	—	990	1600	—	—	—

**Table 3:** Morphological characterization of sorbents.

	CE_diam_ ± SD [μm]	C ± SD	E ± SD	S ± SD
EGDMA + St	130.79 ± 48.30	0.996 ± 0.003	0.006 ± 0.012	1.000 ± 0.003
EGDMA + St + 1L	52.66 ± 32.44	0.992 ± 0.005	0.021 ± 0.023	0.999 ± 0.002
EGDMA + St + 2L	41.86 ± 20.29	0.992 ± 0.005	0.023 ± 0.024	0.999 ± 0.002
EGDMA + St + 3L	30.87 ± 9.33	0.987 ± 0.004	0.047 ± 0.032	0.998 ± 0.002
EGDMA + St + 4L	43.25 ± 23.80	0.990 ± 0.005	0.034 ± 0.029	0.998 ± 0.002
EGDMA + St + 5L	27.91 ± 15.54	0.990 ± 0.005	0.037 ± 0.029	0.999 ± 0.002

**Table 4:** Swelling studies.

Solvent	EGDMA + St	EGDMA + St + 1L	EGDMA + St + 2L	EGDMA + St + 3L	EGDMA + St + 4L	EGDMA + St + 5L	EGDMA + St + 4L-S
	B [%]						
THF	6	8	8	13	27	38	0
ACE	6	13	13	17	27	38	0
ACN	0	5	8	13	27	27	0
TOL	0	15	20	25	25	38	0
TCM	0	6	8	10	17	27	0
MeOH	0	6	6	6	8	8	0
Water	0	0	6	6	8	8	0

**Table 5:** Thermogravimetry data.

Material	T_5%_ (°C)	T_10%_ (°C)	T_50%_ (°C)	T_max1_ (°C)	T_max2_ (°C)	RM (%)
EGDMA + St	286	307	355	358	407	0.32
EGDMA + St + 1L	326	342	376	376	416	1.00
EGDMA + St + 2L	319	337	375	376	—	1.67
EGDMA + St + 3L	319	336	376	377	—	2.52
EGDMA + St + 4L	326	342	379	378	—	3.92
EGDMA + St + 5L	313	328	363	362	—	0.46

**Table 6:** Characteristics of the carbonization process.

Reagents and Concentration	Yield (%)	Carbon Name Acronym (Abbreviation)
Temperature only	8.3%	EGDMA + St + 4L-C (C)
H_3_PO_4_ (85%)	9.1%	EGDMA + St + 4L-P1 (P1)
H_3_PO_4_ (85% diluted in volume ratio 1:1)	10.2%	EGDMA + St + 4L-P2 (P2)
H_2_SO_4_ (95% diluted in volume ratio 1:1)	10.5%	EGDMA + St + 4L-S (S)
HNO_3_ (65% diluted in volume ratio 1:1)	23.9%	EGDMA + St + 4L-N (N)
AgNO_3_ (9%)	26.2%	EGDMA + St + 4L-Ag (Ag)

**Table 7:** Parameters of the porous structures of the studied copolymers.

	Specific Surface Area S_BET_ [m^2^/g]	Total Pore Volume V_TOT_ [cm^3^/g]	Micropore Volume [cm^3^/g]	Average Pore Diameter [nm]
EGDMA + St + 4L	0.55	0.0046	0.0011	40.68
EGDMA + St + 4L-C	12.41	0.0112	0.0064	3.89
EGDMA + St + 4L-P1	4.21	0.0216	0.0008	19.78
EGDMA + St + 4L-P2	21.29	0.0403	0.0013	7.19
EGDMA + St + 4L-S	299.04	0.1492	0.0960	2.00
EGDMA + St + 4L-N	1.02	0.0054	0.0006	22.12
EGDMA + St + 4L-Ag	28.46	0.0309	0.0091	4.45

## Supplementary Materials

The following are available online at https://www.mdpi.com/1996-1944/13/7/1761/s1, Figure S1. The 2D sketch of the 3D particle and circle equivalent idea, Figure S2. Attenuated Total Reflectance Fourier Transform Infrared (ATR-FTIR) spectra of monomers and copolymer of EGDMA + St, Figure S3. Attenuated Total Reflectance Fourier Transform Infrared (ATR-FTIR) spectra of –OH groups in copolymers and original lignin, Figure S4. Attenuated Total Reflectance Fourier Transform Infrared (ATR-FTIR) spectra of carbonized materials. (a) EGDMA + St + L-N, (b) EGDMA + St + L-C, (c) EGDMA + St + L-Ag, (d) EGDMA + St + L-S, (e) EGDMA + St + L-P, (f) EGDMA + St + L-P2, Table S1. ATR-FTIR spectra of carbonized materials.
