# Supplementary material for: Synthesis and Modification by Carbonization of Styrene–Ethylene Glycol Dimethacrylate–Lignin Sorbents and their Sorption of Acetylsalicylic Acid
Source: Materials (Basel). 2020 Apr 9;13(7):1761. doi: 10.3390/ma13071761 (PMC7178683; doi:10.3390/ma13071761)
Supplement: Supplementary file 1 [file materials-13-01761-s001.pdf]

materials

Supplementary

Synthesis and Modification by Carbonization of Styrene–Ethylene Glycol Dimethacrylate–Lignin Sorbents and their Sorption of Acetylsalicylic Acid

Krystyna Wnuczek *, Beata Podkościelna *, Magdalena Sobiesiak, Łukasz Szajnecki and Marta Goliszek

Department of Polymer Chemistry, Institute of Chemical Sciences, Faculty of Chemistry, Maria Curie-Skłodowska University in Lublin, M. Curie-Skłodowska Sq. 2, 20-031 Lublin, Poland; magdalena.sobiesiak@poczta.umcs.lublin.pl (M.S.); l.szajnecki@umcs.pl (Ł.S.); marta.goliszek@poczta.umcs.lublin.pl (M.G.)

* Correspondence: krystyna.wnuczek@poczta.umcs.lublin.pl (K.W.); beatapod@umcs.pl (B.P.)

S2.3. Characterization of methods

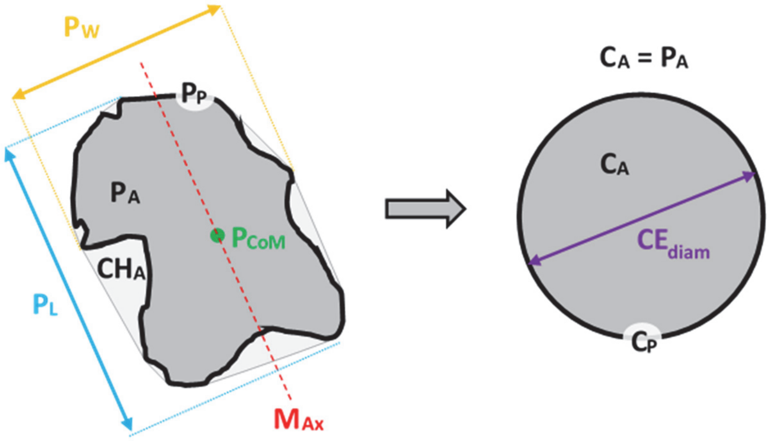

Figure S1. The 2D sketch of the 3D particle and circle equivalent idea.

Three commonly used shape factors are circularity (C), elongation (E) and solidity (S). Circularity (C) is calculated as the ratio of the perimeter of a circle with the same area (C_P) as the particle divided by the perimeter of the particle image (P_P) [41].

$$C = \frac{C_P}{P_P} = \frac{2\times\sqrt{\pi\times P_A}}{P_P} \quad \text{(S1)}$$

Circularity has values in the range 0–1. A circle has a circularity of 1 while a narrow elongated or irregular particle has a circularity value closer to 0. Circularity is sensitive to both elongation and surface roughness.

Elongation (E) of the particle is a parameter calculated as [41]:

$$E = 1 - \frac{P_W}{P_L} \quad \text{(S2)}$$

Elongation takes values in the range 0–1. The higher value of elongation, the more elongated shape of the particle. The particle with a shape symmetrical in all axes (circle, square) has an elongation value of 0 whereas the particles with oblong shapes have an elongation close to 1. Elongation is unaffected by surface roughness.

Solidity of the particle (S) is calculated in accordance to the formula [41]:

$$S = \frac{P_A}{CH_A} \quad (3)$$

Where P_A is the area of the particle and CH_{PA} is the area enclosed by the convex hull. Solidity is the measurement of the overall concavity of a particles and has values in the range 0–1. A smooth shape has a solidity of 1 as the convex hull perimeter is exactly the same as the actual perimeter. When the shape becomes rougher, the solidity value will approach 0.

S3.2. Spectroscopic characterization of copolymers.

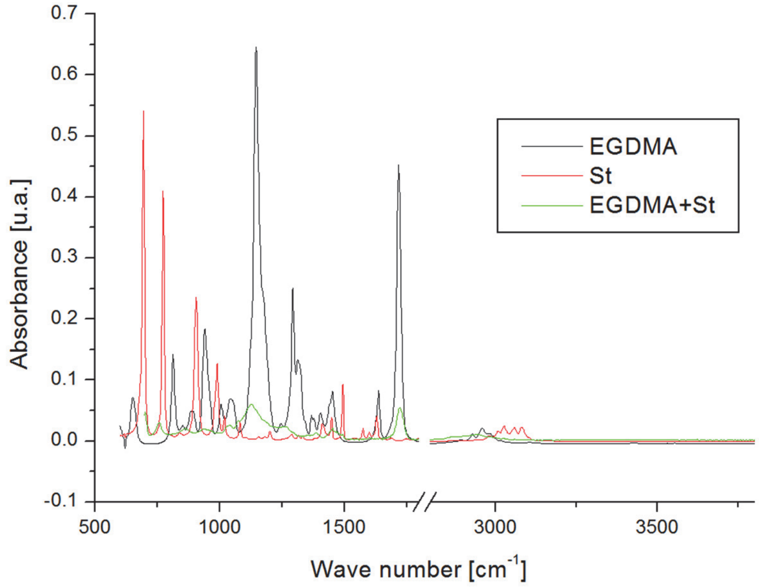

Figure S2. Attenuated Total Reflectance Fourier Transform Infrared (ATR-FTIR) spectra of monomers and copolymer of EGDMA + St.

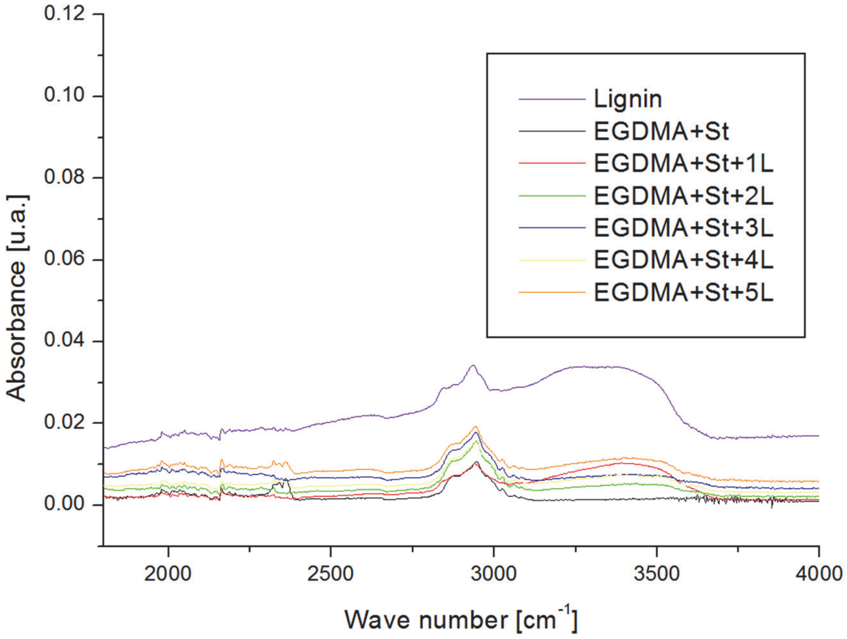

Figure S3. Attenuated Total Reflectance Fourier Transform Infrared (ATR-FTIR**)** spectra of –OH groups in copolymers and original lignin.

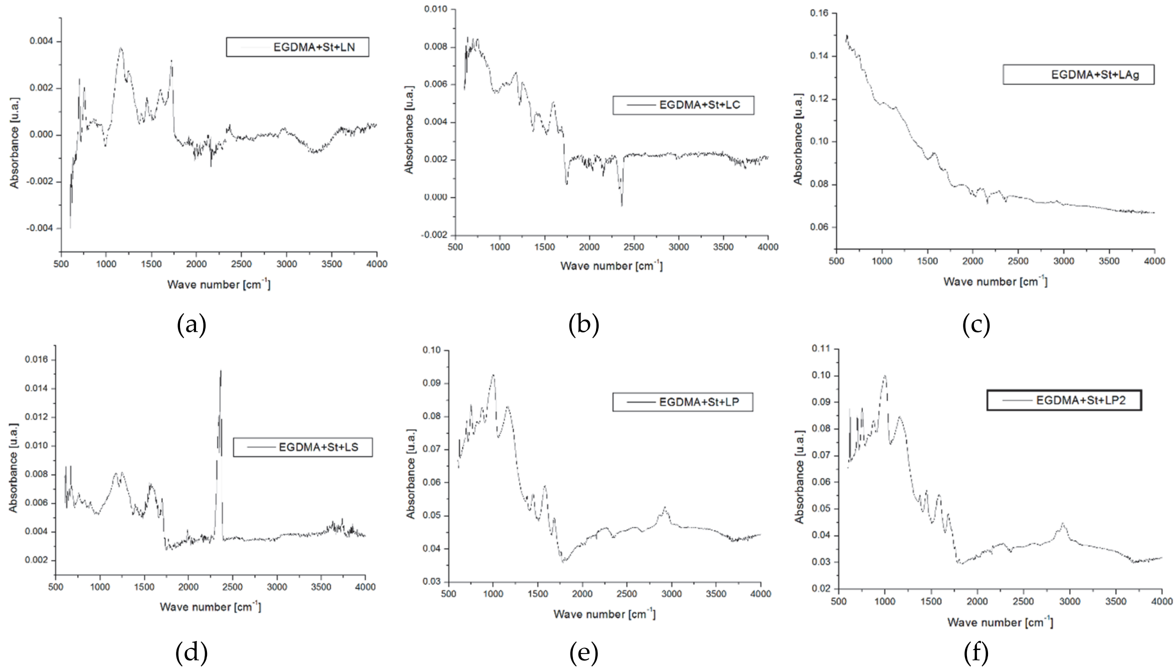

Figure S4. Attenuated Total Reflectance Fourier Transform Infrared (ATR-FTIR) **s**pectra of carbonized materials. (**a**) EGDMA + St + L-N, (**b**) EGDMA + St + L-C, (**c**) EGDMA + St + L-Ag, (**d**) EGDMA + St + L-S, (**e**) EGDMA + St + L-P, (**f**) EGDMA + St + L-P2.

Table S1. ATR-FTIR spectra of carbonized materials.

Material	Signal [cm^{-1}]	Characteristic Group
EGDMA + St + L-N	700	C-H (arom.)
	1600	N=O
	1720	Carboxylic acid,lactone
EGDMA + St + L-Ag	—	—
EGDMA + St + L-S	616	SO_2 (sym.)
	1247	SO_2 (asym.)
	1700	C=O
EGDMA + St + L-P<br>EGDMA + St + L-P2	620	P-O-Ar
	750	P-O-C (sym.)
	873	P-CH_3
	998	P-O-C
	1158	P=O
EGDMA + St + L-C	613	C-H
	698	CH_2
	753	-O-CH_3
	1173	C-O-C
	1595	Anhydrate/lacton
